# Heterogeneity in academic burnout and its association with professional identity among undergraduate nursing students: a latent profile analysis

**DOI:** 10.3389/fpubh.2026.1875927

**Published:** 2026-07-07

**Authors:** Mingfen Tao, Xinyue Chen, Wanyu Ding, Yingying Wang, Shaoyong Ma

**Affiliations:** 1 Department of Nursing, The First Affiliated Hospital of Wannan Medical University, Wuhu, Anhui, China; 2 Graduate School, Wannan Medical University, Wuhu, Anhui, China; 3 Emergency Intensive Care Unit, The First Affiliated Hospital of Wannan Medical University, Wuhu, Anhui, China; 4 School of Nursing, Wannan Medical University, Wuhu, Anhui, China

**Keywords:** academic burnout, latent profile analysis, nursing education, nursing students, professional identity, public health

## Abstract

**Objectives:**

To identify latent profiles of academic burnout among undergraduate nursing students and examine the demographic, academic, and professional-identity differences across these profiles.

**Methods:**

A cross-sectional survey was conducted from October to December 2025 among 741 undergraduate nursing students from four universities in Anhui Province, China. Data were collected using a demographic questionnaire, the Learning Burnout of University Students scale, and the Professional Identity Scale. Latent profile analysis was performed using the 20 academic-burnout items as manifest indicators. Model selection was based on information criteria, entropy, likelihood-ratio tests, profile size, posterior classification probabilities, parsimony, and interpretability. Chi-square tests and one-way analysis of variance were used for descriptive profile comparisons. To complement statistical screening with conceptual considerations, factors associated with profile membership were examined using an exploratory multinomial logistic regression model informed by a conceptual framework based on directed acyclic graph principles.

**Results:**

Three profiles were identified: low academic burnout (*n* = 132, 17.81%), moderate academic burnout (*n* = 381, 51.42%), and high academic burnout (*n* = 228, 30.77%). The three profiles mainly represented a low-to-high severity gradient rather than qualitatively distinct burnout patterns. Compared with the low-burnout profile, students with poorer academic performance were more likely to be assigned to the moderate- or high-burnout profiles. Sophomore students had higher odds of being in the high-burnout profile than senior students, whereas confidence in employment prospects was associated with lower odds of high burnout. Professional identity scores decreased progressively from the low- to the high-burnout profile, with statistically significant differences across all pairwise comparisons.

**Conclusion:**

Academic burnout among undergraduate nursing students is heterogeneous, and approximately one-third of students were classified into a high-burnout profile. Professional identity showed a clear inverse gradient across burnout profiles. In the conceptual-framework-informed exploratory regression model, academic performance, grade level, only-child status, reasons for choosing nursing, and perceived employment prospects were associated with profile membership. Because of the cross-sectional design, possible residual confounding, and classification uncertainty, these associations should not be interpreted as causal. Nursing educators and public health education administrators should consider profile-specific strategies that combine academic support, career guidance, psychological support, and professional identity development.

## 1 Introduction

Burnout is defined as a chronic stress-induced psychological syndrome marked by exhaustion, disengagement and reduced efficacy (1). In student populations, this manifests as academic burnout: learning-related emotional exhaustion, negative attitudes toward study and diminished academic achievement (2). Academic burnout is not only an educational problem but also a public health issue, because it is associated with psychological distress, poor learning engagement, lower academic performance, and potential withdrawal from training (3).

The issue is particularly important in nursing education. As future nursing practitioners, undergraduate students’ learning experiences may shape their professional socialization, career commitment, and clinical practice. Previous studies in China have reported relatively high levels of academic burnout among nursing students (4, 5). Amid growing demand for competent nursing staff, early detection of high-burnout students enables institutions to deliver preventive support prior to clinical placement.

Professional identity serves as a critical protective factor against academic burnout among nursing students (6, 7). While existing studies have widely confirmed this negative association, current evidence remains largely limited to descriptive analyses conducted only at the overall group level. Most previous studies adopt variable-centered approaches that assume population homogeneity, thereby ignoring individual differences and failing to explore whether the protective effect of professional identity varies across distinct burnout subgroups. This leaves an evident research gap for developing targeted educational interventions.

Despite growing research on academic burnout in nursing education, most studies adopt variable-centered approaches, such as regression or structural equation modeling, that assume population homogeneity and overlook meaningful subgroup differences (8, 9). Evidence concerning Chinese undergraduate nursing students remains especially limited, despite their diverse burnout-related traits and distinct learning needs. A person-centered method such as latent profile analysis (LPA) can identify unobserved subgroups with distinct academic burnout patterns, offering more actionable insights for targeted and personalized nursing education interventions (10–12). Therefore, this study used LPA to identify academic burnout profiles among undergraduate nursing students and explore subgroup differences in demographic, academic performance, and professional-identity to inform profile-specific academic support, career guidance, and professional identity interventions in nursing education.

## 2 Materials and methods

### 2.1 Study design and reporting

This cross-sectional study was conducted from October to December 2025. The reporting of the study was guided by the Strengthening the Reporting of Observational Studies in Epidemiology (STROBE) statement for cross-sectional studies (13).

### 2.2 Setting and participants

Participants were recruited from four universities in Anhui Province, China, using convenience sampling. This cross-sectional study recruited 741 undergraduate nursing students from one provincial key university and three provincial general universities. These universities differ in location, academic focus, faculty strength, and student demographics, resulting in variation in learning atmosphere and emphasis on professional identity cultivation. Eligible participants were full-time undergraduate nursing students who provided informed consent and voluntarily agreed to participate. Students who were not on campus during the survey period, such as those on leave or suspension, and those with a current or previous diagnosis of mental illness were excluded. The exclusion criterion related to diagnosed mental illness was used to reduce potential confounding by severe pre-existing mental health conditions; however, its implications for representativeness are addressed in the limitations section. Data were collected using an online survey platform.^1^ The recruitment process was as follows: The research team contacted the academic affairs office of each school of nursing and obtained official approval. Subsequently, questionnaire links were released in class groups and forwarded by counselors or class monitors, and students filled out the questionnaires voluntarily.

### 2.3 Sample size

For multivariable analyses, the minimum sample size was estimated using the common rule of 5–10 participants per independent variable. Fourteen independent variables were considered, yielding a minimum requirement of 70–140 participants; after allowing for a 20% invalid response rate, the minimum target was 88–175 participants (14). Because latent profile analysis generally requires larger samples to obtain stable profile solutions, a sample size above 300 was considered desirable (10). A total of 806 questionnaires were collected, and 741 valid questionnaires were included in the final analysis, exceeding both requirements. A post-hoc power analysis was performed using G*Power 3.1. With a two-sided significance level of *α* = 0.05 and a small-to-medium effect size (odds ratio = 1.3), the final sample of 741 participants achieved a statistical power of 0.885, exceeding the recommended threshold of 0.80.

### 2.4 Measures

#### 2.4.1 Demographic and academic characteristics

A researcher-designed questionnaire was used to collect information on gender, grade level, place of household registration, reason for choosing the nursing major, only-child status, student cadre experience, academic performance, paternal education level, maternal education level, attitude toward the nursing major, parental support for the major, monthly household income, perceived school learning environment, and perceived employment prospects of the nursing major.

#### 2.4.2 Learning burnout of university students scale

Academic burnout was assessed using the Learning Burnout of University Students scale developed by Lian et al. (15). The scale contains 20 items across three dimensions: depression, misbehavior, and low sense of achievement. Items are scored on a 5-point Likert scale ranging from 1 (completely inconsistent) to 5 (completely consistent), with higher scores indicating higher academic burnout. The original scale showed good internal consistency, and the Cronbach’s alpha coefficient in the present study was 0.840 (95% CI: 0.817–0.860).

#### 2.4.3 Professional identity scale

Professional identity was measured using the Professional Identity Scale developed by Qin (7). The scale includes 23 items across four dimensions: cognitive identity, emotional identity, behavioral identity, and appropriateness identity. Items are rated on a 5-point Likert scale, with higher scores indicating stronger professional identity. The Cronbach’s alpha coefficient was 0.916 in the original study and 0.909 (95% CI: 0.896–0.921) in the present study.

### 2.5 Data collection and quality control

After permission was obtained from the relevant university departments, members of the research team explained the study purpose, voluntary nature of participation, confidentiality protection, and questionnaire procedures to eligible students. An online questionnaire link was then distributed to students who agreed to participate. The questionnaire was set to require completion of all items before submission, and each student could submit the questionnaire only once. A total of 806 questionnaires were returned. Responses with obvious regular response patterns or implausible completion times (<240 s or >720 s, according to the prespecified quality-control rule) were excluded. Finally, 741 valid questionnaires were retained, yielding an effective response rate of 91.94%.

### 2.6 Statistical analysis

Statistical analyses were performed using Mplus 8.3 and SPSS 26.0. Descriptive statistics were used to summarize participant characteristics and scale scores. Continuous variables were presented as mean ± standard deviation (SD), and categorical variables were presented as frequency and percentage.

Latent profile analysis (LPA) was performed in Mplus 8.3. The 20 items of the academic burnout scale were adopted as continuous manifest indicators. We sequentially estimated models with 1 to 5 latent profiles. To ensure stable model convergence, 1,300 random starting solutions and 250 final stage optimizations were set for model iteration. The maximum likelihood (ML) estimation method was applied. Since the questionnaire was set to be fully completed before submission, there was no missing data in the final valid dataset, so no missing data processing procedure was required.

Model selection was based on the Akaike information criterion (AIC), Bayesian information criterion (BIC), sample-size-adjusted BIC (aBIC), entropy, Lo–Mendell–Rubin adjusted likelihood ratio test (LMR-LRT), bootstrapped likelihood ratio test (BLRT), profile proportions, average posterior probabilities, model parsimony, and substantive interpretability. Lower AIC, BIC, and aBIC values indicate better relative fit; entropy values closer to 1 indicate better classification accuracy; and significant LMR-LRT and BLRT values suggest that the k-profile model fits significantly better than the k-1 profile model (10, 16, 17).

After the optimal profile solution was selected, participants were assigned to their most likely profile according to maximum posterior probabilities. Chi-square tests and one-way analysis of variance (ANOVA) were used for descriptive comparisons across profiles.

To address the limitation of relying solely on univariate statistical significance for variable selection, the measured covariates were additionally organized within a conceptual framework informed by directed acyclic graph (DAG) principles and methodological literature on confounder selection (18–21). The framework included three conceptual domains: demographic background, family socioeconomic and support factors, and educational/professional-context factors. Demographic background included gender, grade level, place of household registration, and only-child status. Family socioeconomic and support factors included paternal education level, maternal education level, monthly household income, and parental support for the nursing major. Educational/professional-context factors included reason for choosing nursing, student cadre experience, academic performance, attitude toward nursing, perceived school learning environment, and perceived employment prospects.

Because this was an exploratory cross-sectional study and several variables had multiple categories, the multinomial logistic regression model used a pragmatic strategy that combined conceptual plausibility with descriptive profile differences. Variables that were conceptually relevant and statistically different across latent profiles in univariate analyses were entered into the exploratory multinomial logistic regression model. The low academic burnout profile was used as the reference category, and odds ratios (ORs) with 95% confidence intervals (CIs) were reported. The regression results were interpreted as exploratory adjusted associations rather than causal effects, because residual confounding and reverse causality could not be excluded.

Professional identity was not entered as an adjustment covariate in the multinomial logistic regression model because it was the main external characteristic compared across burnout profiles, and its temporal ordering relative to academic burnout could not be determined in the cross-sectional design. A two-sided *p* value <0.05 was considered statistically significant. Because the available dataset was analyzed using maximum posterior probability assignment for subsequent comparisons, the possibility of classification-error bias could not be fully eliminated. Therefore, the multinomial regression and ANOVA findings should be interpreted cautiously.

### 2.7 Ethical considerations

The study protocol was approved by the Ethics Committee of the First Affiliated Hospital of Wannan Medical University (approval number: LL-2024BH03) and complied with the Declaration of Helsinki. All participants provided informed consent before completing the questionnaire. Participation was voluntary, and all data were collected anonymously and used only for research purposes.

## 3 Results

### 3.1 Participant characteristics and scale scores

A total of 741 undergraduate nursing students were included. The total academic burnout score was 54.34 ± 9.65, and the average item score was 2.72 ± 0.48. The total professional identity score was 82.48 ± 10.92, and the average item score was 3.59 ± 0.48.

### 3.2 Latent profiles of academic burnout

**Figure 1:**
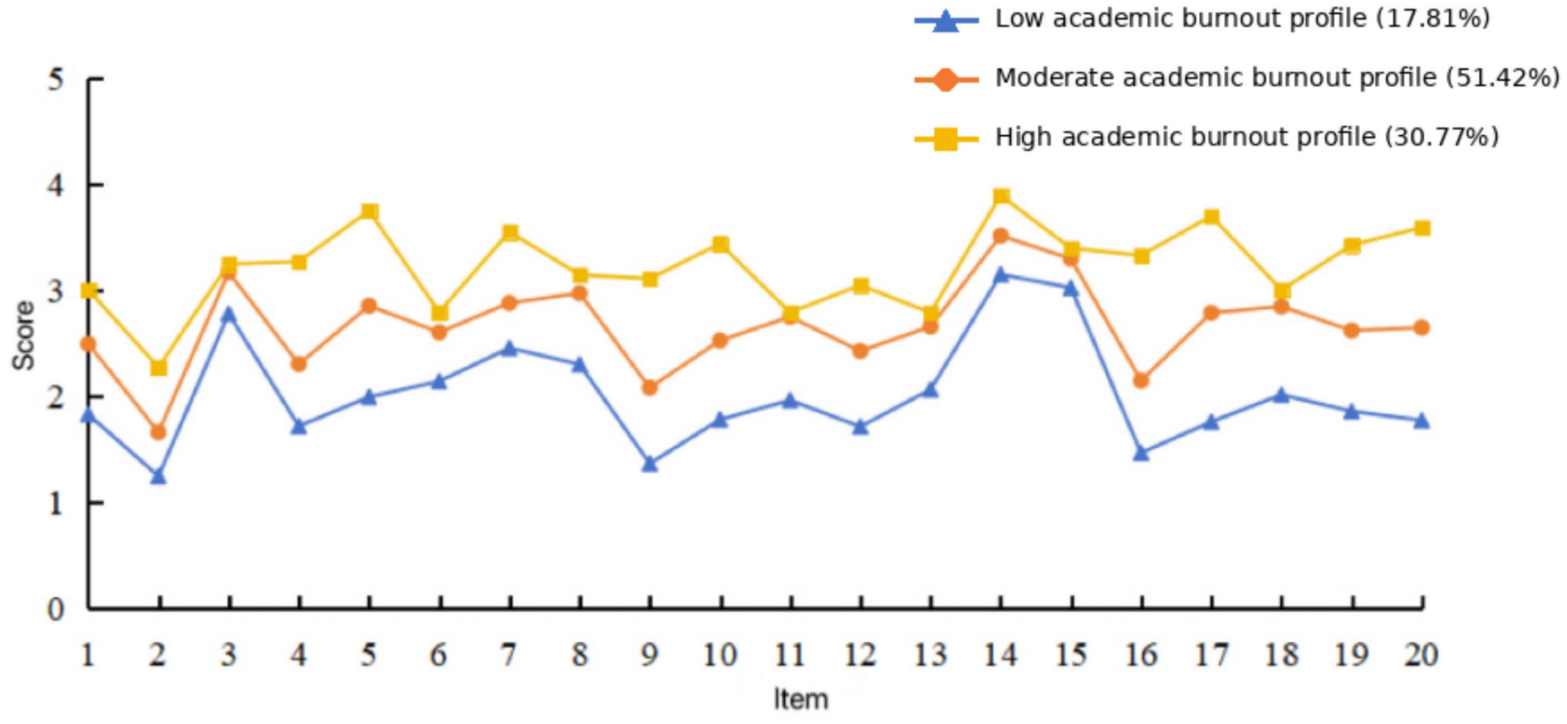
Profile plot of the three latent academic-burnout profiles across the 20 manifest indicators. C1, low academic burnout; C2, moderate academic burnout; C3, high academic burnout.

The fit indices for the one- to five-profile models are shown in Table 1. The AIC, BIC, and aBIC values decreased as the number of profiles increased. The five-profile model had a non-significant LMR-LRT value (*p* > 0.05), suggesting that it did not improve significantly over the four-profile model. Although the four-profile model showed the highest entropy, one profile accounted for less than 10% of the sample, reducing its practical interpretability. The three-profile model had acceptable entropy (0.824), significant LMR-LRT and BLRT values, profile proportions above 10%, and high average posterior probabilities (0.917–0.923). Therefore, the three-profile solution was selected as the optimal model (Figure 1; Table 2).

**Table 1:** Model-fit indices for latent profile models of academic burnout among undergraduate nursing students (*n* = 741).

Model	AIC	BIC	aBIC	Entropy	LMR-LRT *p*	BLRT *p*	Class proportions (%)
1-profile	40995.190	41179.510	41052.495	-	-	-	-
2-profile	39127.801	39408.889	39215.192	0.843	<0.001	<0.001	49.2/50.8
3-profile	38671.725	39049.581	38789.202	0.824	0.031	<0.001	17.8/51.4/30.8
4-profile	38333.358	38807.982	38480.920	0.856	0.021	<0.001	17.5/7.2/52.5/22.8
5-profile	38122.579	38693.971	38300.227	0.836	0.338	<0.001	13.2/36.1/36.4/7.1/7.2

AIC, Akaike information criterion; BIC, Bayesian information criterion; aBIC, sample-size-adjusted Bayesian information criterion; LMR-LRT, Lo–Mendell–Rubin adjusted likelihood ratio test; BLRT, bootstrapped likelihood ratio test.

**Table 2:** Average posterior probabilities for the three-profile solution (*n* = 741).

Assigned profile	C1	C2	C3
C1: Low academic burnout	0.923	0.077	0.000
C2: Moderate academic burnout	0.035	0.920	0.046
C3: High academic burnout	0.000	0.083	0.917

The three profiles were labeled according to their score levels as low, moderate and high academic burnout profile. The three profiles showed a consistent low-to-high severity gradient across the total academic burnout score and its three dimensions. Notably, these profiles only differ in the intensity of academic burnout rather than presenting qualitatively distinct burnout patterns, so the results should be interpreted with caution (Table 3).

**Table 3:** Academic burnout dimension scores across the three latent profiles (mean ± SD).

Dimension	Total sample (*n* = 741)	C1 (*n* = 132)	C2 (*n* = 381)	C3 (*n* = 228)	F	*p*	η^2^
Depression	20.69 ± 5.28	13.83 ± 2.66	19.76 ± 3.05	26.21 ± 3.47	692.37	<0.001	0.652
Misbehavior	16.79 ± 3.63	12.32 ± 2.03	16.31 ± 2.39	20.18 ± 2.74	447.03	<0.001	0.548
Low sense of achievement	16.87 ± 3.19	13.95 ± 2.54	17.26 ± 2.64	17.89 ± 3.39	85.81	<0.001	0.189
Total academic burnout	54.34 ± 9.65	40.11 ± 4.67	53.33 ± 4.55	64.28 ± 6.10	955.76	<0.001	0.721

### 3.3 Univariate comparisons across latent profiles

Univariate analyses showed statistically significant differences across the three profiles in grade level, reason for choosing the nursing major, only-child status, student cadre experience, academic performance, attitude toward the nursing major, perceived school learning environment, and perceived employment prospects of the nursing major (all *p* < 0.05). Gender, place of household registration, parental education level, parental support for the major, and monthly household income were not significantly different across profiles (Table 4).

**Table 4:** Univariate comparisons of participant characteristics across the three latent profiles (*n* = 741).

Variable	Total	C1	C2	C3	χ^2^	*p*	Cramer’s V
Gender: male/female	172/569	33/99	81/300	58/170	1.686	0.431	0.048
Grade: freshman/sophomore/junior/senior	260/198/139/144	60/19/28/25	142/76/75/88	58/103/36/31	62.033	<0.001	0.205
Place of registration: rural/township/county/city	435/97/118/91	71/18/26/17	236/49/55/41	128/30/37/33	4.596	0.557	0.057
Reason for choosing nursing: interest/parents/relatives/teacher/adjustment/other	151/147/83/20/177/163	37/15/14/5/39/22	77/76/39/13/93/83	37/56/30/2/45/58	24.707	0.006	0.129
Only child: yes/no	182/559	45/87	74/307	63/165	13.059	<0.001	0.133
Student cadre experience: yes/no	159/582	41/91	79/302	39/189	9.904	0.007	0.116
Academic performance: good/medium/average	79/384/278	28/77/27	34/203/144	17/104/107	35.626	<0.001	0.155
Father education: illiterate/primary/junior/senior/college/above bachelor	16/193/344/120/54/14	5/26/62/23/13/3	4/103/183/56/30/5	7/64/99/41/11/6	13.682	0.188	0.096
Mother education: illiterate/primary/junior/senior/college/above bachelor	57/290/273/81/32/8	9/51/48/15/6/3	29/149/143/42/14/4	19/90/82/24/12/1	3.900	0.952	0.051
Attitude toward nursing: like/neutral/dislike	247/440/54	62/63/7	130/230/21	55/147/26	25.229	<0.001	0.130
Parental support: support/neutral/not support	502/211/28	96/32/4	256/113/12	150/66/12	5.872	0.209	0.049
Monthly household income: 2000 CNY	75/149/136/123/258	9/19/32/27/45	38/83/64/67/129	28/47/40/29/84	12.127	0.146	0.090
School learning environment: good/average/poor	280/396/65	75/53/4	142/213/26	63/130/35	42.102	<0.001	0.169
Employment prospects: confident/neutral/indifferent	296/345/100	83/40/9	155/180/46	58/125/45	51.832	<0.001	0.187

Values are n unless otherwise indicated. C1, low academic burnout; C2, moderate academic burnout; C3, high academic burnout.

These univariate comparisons were used to describe the characteristics of the latent profiles and to identify candidate variables for the exploratory regression model; they were not interpreted as evidence of causal relationships.

### 3.4 Factors associated with latent profile membership

Based on the conceptual-framework-informed exploratory modeling strategy described above, variables that were both conceptually plausible and descriptively different across latent profiles were entered into the multinomial logistic regression model. The coding scheme and reference categories are shown in Table 5. The low academic burnout profile (C1) was used as the reference outcome category. The subgroup of participants who selected the nursing major based on teacher recommendation comprised merely 20 individuals; therefore, the corresponding regression estimates may be unstable and should be interpreted with caution.

**Table 5:** Conceptual domains and coding of variables entered into the exploratory multinomial logistic regression model.

Conceptual domain	Variable	Dummy-variable setting	Reference category
Demographic background	Grade	Freshman, sophomore, junior	Senior
Demographic background	Only-child status	Yes	No
Educational/professional context	Reason for choosing nursing	Personal interest, parental request, relative recommendation, teacher recommendation, major adjustment	Others
Educational/professional context	Student cadre experience	Yes	No
Educational/professional context	Academic performance	Good, medium	Average
Educational/professional context	Attitude toward nursing	Like, neutral	Dislike
Educational/professional context	School learning environment	Good, average	Poor
Educational/professional context	Employment prospects	Confident, neutral	Indifferent

Family socioeconomic/support variables were considered in the conceptual framework but were not entered into the exploratory regression model because they did not show descriptive differences across profiles in the present dataset. The regression model therefore used a pragmatic strategy that combined conceptual plausibility with descriptive profile differences.

The regression results indicated that only-child status, academic performance, grade level, reason for choosing the nursing major, and perceived employment prospects were significantly associated with profile membership (Table 6). The Nagelkerke pseudo R^2^ of the multinomial logistic model was 0.229. VIF values of all independent variables ranged from 1.019 to 1.342 (all < 5), suggesting no multicollinearity. The Hosmer-Lemeshow χ^2^ = 7.50 (df = 8, *p* = 0.484), and AUC = 0.730 (95% CI: 0.694–0.766), showing good calibration and acceptable discriminative ability. Because the model was based on cross-sectional data and used an exploratory variable-selection strategy, these findings should be interpreted as adjusted associations rather than causal effects.

**Table 6:** Multinomial logistic regression for profile membership.

Comparison	Variable	Reference	β	SE	Wald χ^2^	*p*	OR (95% CI)
C2 vs. C1	Only child: yes	No	−0.679	0.249	7.421	0.006	0.507 (0.311–0.827)
C2 vs. C1	Good academic performance	Average	−1.358	0.356	14.549	<0.001	0.257 (0.128–0.517)
C2 vs. C1	Medium academic performance	Average	−0.593	0.260	5.188	0.023	0.553 (0.332–0.921)
C3 vs. C1	Sophomore grade	Senior	0.898	0.419	4.598	0.032	2.455 (1.080–5.581)
C3 vs. C1	Teacher recommendation	Others	−1.937	0.904	4.587	0.032	0.144 (0.025–0.848)
C3 vs. C1	Good academic performance	Average	−1.848	0.418	19.561	<0.001	0.158 (0.070–0.357)
C3 vs. C1	Medium academic performance	Average	−0.789	0.284	7.702	0.006	0.455 (0.260–0.793)
C3 vs. C1	Confident employment prospects	Indifferent	−1.364	0.452	9.101	0.003	0.256 (0.105–0.620)

C1, low academic burnout; C2, moderate academic burnout; C3, high academic burnout. Reference categories for predictors: only-child status = no; grade = senior; reason for choosing nursing = others; academic performance = average; employment prospects = indifferent. Variables were selected using a pragmatic strategy that combined conceptual plausibility with descriptive profile differences. All variables listed in Table 5 were entered into the exploratory multinomial logistic regression model; only statistically significant predictors are presented in this table for readability. The results should be interpreted as exploratory adjusted associations rather than causal effects.

### 3.5 Professional identity across latent profiles

Professional identity differed significantly across the three academic-burnout profiles. Total professional identity and all four dimensions showed a decreasing gradient from C1 to C3 (all *p* < 0.001). Post-hoc comparisons indicated that all pairwise differences were statistically significant, with C1 showing the highest professional identity and C3 the lowest (Table 7).

**Table 7:** Professional identity scores across the three latent profiles (mean ± SD).

Profile	n	Total score	Cognitive identity	Emotional identity	Behavioral identity	Appropriateness identity
C1: Low academic burnout	132	91.64 ± 10.64	20.78 ± 2.68	31.98 ± 5.28	24.60 ± 3.15	14.29 ± 2.81
C2: Moderate academic burnout	381	82.36 ± 9.96	19.15 ± 2.73	28.97 ± 5.47	21.61 ± 2.94	12.63 ± 2.50
C3: High academic burnout	228	77.37 ± 12.55	18.66 ± 2.86	27.07 ± 5.95	19.78 ± 3.74	11.87 ± 3.16
F		71.23	25.66	32.32	92.69	31.98
P		<0.001	<0.001	<0.001	<0.001	<0.001
η^2^		0.162	0.065	0.081	0.201	0.080
Post-hoc test		C1 > C2 > C3	C1 > C2 > C3	C1 > C2 > C3	C1 > C2 > C3	C1 > C2 > C3

LSD test for cognitive and emotional identity; Tamhane’s T2 test for behavioral identity, appropriateness, and total score.

## 4 Discussion

### 4.1 Principal findings

This study identified three latent profiles of academic burnout among undergraduate nursing students: low, moderate, and high academic burnout. More than half of the students were assigned to the moderate-burnout profile, and nearly one-third were assigned to the high-burnout profile. The profiles represented a severity gradient rather than qualitatively distinct response patterns. This finding suggests that academic burnout among nursing students may be best understood as a hierarchical risk continuum in this sample. Such a continuum is still meaningful for practice because it can help educators identify students who require different levels of academic, psychological, and career-development support.

### 4.2 Interpretation of the three academic-burnout profiles

Students in the low-burnout profile reported lower levels of depression, misbehavior, and low sense of achievement, suggesting relatively adaptive learning states. Students in the moderate-burnout profile represented the largest subgroup and may be at a transitional stage in which timely support could prevent escalation. Students in the high-burnout profile reported elevated scores across all dimensions, indicating a need for more intensive and integrated support. Because the three profiles differed mainly by severity, future studies should explore whether additional psychological, motivational, or contextual indicators can identify more qualitatively distinct burnout patterns.

### 4.3 Factors associated with profile membership

The factors associated with profile membership should be interpreted as exploratory adjusted associations rather than causal determinants. To address the limitation of selecting variables solely according to univariate statistical significance, the revised analysis clarified that the candidate variables were considered within a conceptual framework informed by DAG principles. This framework grouped measured covariates into demographic background, family socioeconomic/support factors, and educational/professional-context factors. Nevertheless, because the study was cross-sectional and the regression model was not designed to estimate the causal effect of a single exposure, residual confounding and reverse causality cannot be excluded.

Only-child status was associated with profile membership. Compared with non-only children, only children had lower odds of being assigned to the moderate-burnout profile than to the low-burnout profile. This finding differs from some previous studies reporting no significant differences between only-child and non-only-child students (22). One possible explanation is that only children may receive more concentrated family resources and academic support. Nevertheless, this interpretation is not supported by empirical data in the current study, as we did not collect quantitative information on family functioning, perceived social support, or household economic conditions. Therefore, we cannot confirm the causal pathway between family resources and burnout profiles. This explanation is merely a reasonable deduction based on existing literature rather than a conclusion drawn from our own measurements (23–25). Future research needs to incorporate these variables for further validation.

Academic performance was one of the most consistent factors associated with profile membership. Students with good or medium academic performance were less likely to belong to the moderate- or high-burnout profiles than students with average academic performance. This finding is consistent with prior research suggesting that academic difficulties are closely related to burnout among medical and nursing students (26, 27). Poorer academic performance may increase frustration and reduce academic self-efficacy, while burnout may in turn impair concentration and learning efficiency, creating a reciprocal negative cycle. Therefore, early academic tutoring and learning-skills training may be important components of burnout prevention.

Grade level was also associated with high academic burnout. Sophomore students had higher odds of being in the high-burnout profile than senior students. This may reflect the transition from general foundational courses to more specialized and demanding nursing courses, including laboratory training and early professional socialization. Students at this stage may experience increased academic pressure while still lacking a stable professional identity and effective coping strategies (28). Nursing programs should therefore consider grade-specific monitoring and support, particularly during the sophomore year.

The reason for choosing the nursing major was another relevant factor. Students who chose nursing based on teacher recommendation were less likely to belong to the high-burnout profile than those who selected “other” reasons. However, this result should be interpreted cautiously because the teacher-recommendation subgroup was small, which may lead to unstable statistical estimates. We tentatively speculate that professional guidance from teachers can help students acquire objective and comprehensive knowledge about nursing before enrollment, reduce unrealistic career expectations, and improve their psychological adaptation to the major (29). It must be emphasized that this is only a mechanistic conjecture. We did not assess students’ professional cognition, expectation level and adaptation status in this survey, so the above pathway cannot be empirically tested. In view of this, we present this explanation cautiously and regard it as a reference for subsequent exploration rather than a definitive finding. Pre-enrollment professional orientation and realistic career information may help students make more informed choices and reduce subsequent burnout risk.

Perceived employment prospects were strongly associated with burnout profiles. Students who were confident about employment prospects were less likely to be assigned to the high-burnout profile. Recent studies similarly suggest that future professional prospects are an important factor related to academic burnout among undergraduate nursing students (30). Positive career expectations may strengthen learning purpose, enhance persistence, and help students interpret current academic demands as meaningful preparation for future work (31, 32). Career-planning courses, alumni mentoring, and transparent information about career pathways may therefore be useful components of burnout prevention.

### 4.4 Relationship between academic burnout profiles and professional identity

Professional identity showed a clear inverse gradient across burnout profiles: students in the low-burnout profile had the highest professional identity, whereas those in the high-burnout profile had the lowest. This result supports the view that professional identity and academic burnout are closely related in nursing education. Stronger professional identity is associated with a clearer sense of professional meaning, greater learning motivation, and stronger behavioral commitment to the nursing profession. Conversely, higher academic burnout is associated with a weaker sense of belonging and reduced willingness to invest effort in professional learning. Because the present study was cross-sectional, the directionality of this relationship cannot be determined. Longitudinal studies are needed to test whether professional identity protects against later burnout, whether burnout erodes professional identity, or whether both processes occur simultaneously.

### 4.5 Implications for nursing education and public health practice

The findings have several practical implications. First, nursing schools should establish early screening systems for academic burnout, with particular attention to students in the moderate- and high-burnout profiles. Second, support should be tailored to profile-specific needs: students with moderate burnout may benefit from academic tutoring, time-management training, and peer support, whereas students with high burnout may require integrated academic counseling, psychological support, and close follow-up. Third, professional identity development should be embedded throughout nursing education. Strategies may include clinical role-model mentoring, reflective learning, realistic career guidance, and structured exposure to diverse nursing career pathways. Systematic reviews suggest that interventions such as cognitive-behavioral strategies, relaxation, peer discussion, and mindfulness-based approaches may help reduce burnout among nursing students (33). From a public health perspective, reducing academic burnout and strengthening professional identity may contribute to a healthier and more stable future nursing workforce.

### 4.6 Strengths and limitations

This study has several strengths. It used a relatively large sample of undergraduate nursing students from four universities and applied a person-centered method to identify heterogeneity in academic burnout. It also examined professional identity differences across profiles, providing practical information for targeted interventions.

Several limitations should be acknowledged. First, the cross-sectional design precludes causal inference. Second, convenience sampling from universities in a single Chinese province limits generalizability. Third, participants were nested within different universities, but we did not explicitly model the hierarchical structure of the data. Students within the same university may share similar learning environments, curricula, and institutional support systems, which could introduce intra-class correlation and potentially bias standard errors. The absence of multilevel modeling (e.g., mixed-effects models) to account for this nested structure is a methodological limitation. Future research should employ hierarchical analytic approaches to address this clustering effect and obtain more accurate estimates. Fourth, all data were collected using self-report questionnaires, which may introduce common-method and social-desirability bias. Fifth, we excluded participants with a history of mental illness to control for confounding factors. However, this group tends to report higher levels of academic burnout, so their exclusion may bias the results and underestimate the true prevalence and severity of burnout in nursing undergraduates. Consequently, the generalizability of our findings to nursing students with a history of mental illness is limited, and future studies are encouraged to include this population to test the robustness of our results. Sixth, although the variable-selection strategy was complemented by a conceptual framework informed by DAG principles, the regression model remained exploratory. Some measured variables that were conceptually relevant but not statistically different in univariate comparisons were not included in the final regression model, which may have resulted in residual confounding. In addition, several potentially important factors, such as anxiety, depression, resilience, perceived social support, academic stress, sleep quality, coping style, and clinical-practice pressure, were not measured. Seventh, although the three-profile model showed acceptable classification quality, subsequent analyses used maximum posterior probability assignment and did not fully account for classification error. Future studies should apply three-step methods such as R3STEP and BCH, and should conduct longitudinal, multicenter studies to verify the stability and predictive validity of the identified profiles.

## 5 Conclusion

Undergraduate nursing students in this study showed three latent profiles of academic burnout: low, moderate, and high burnout. The profiles mainly reflected a severity gradient, and students in higher-burnout profiles exhibited progressively lower professional identity scores in this cross-sectional sample. In the conceptual-framework-informed exploratory regression model, academic performance, grade level, only-child status, reasons for choosing nursing, and perceived employment prospects were associated with profile membership. Because of the cross-sectional design, possible residual confounding, and classification uncertainty, these associations should not be interpreted as causal, and the temporal direction between professional identity and academic burnout cannot be determined. Nursing educators should identify students at different burnout-risk levels and provide tiered interventions that integrate academic support, career guidance, psychological support, and professional identity development. Longitudinal studies are needed to assess the stability of the identified profiles over time and to examine the evolution of academic burnout.

## Data Availability

The original contributions presented in the study are included in the article/supplementary material, further inquiries can be directed to the corresponding authors.

## Footnotes

1
https://www.wjx.cn/
